# A modified Delphi to define drug dosing errors in pediatric critical care

**DOI:** 10.1186/s12887-020-02384-3

**Published:** 2020-10-21

**Authors:** Nadia Roumeliotis, Eleanor Pullenayegum, Paula Rochon, Anna Taddio, Chris Parshuram

**Affiliations:** 1 Department of Critical Care Medicine, The Hospital for Sick Children, 555 University Avenue, Toronto, ON Canada; 2 Child Health Evaluative Sciences, SickKids Research Institute, Toronto, Canada; 3 Dalla Lana School of Public Health, University of Toronto, Toronto, ON Canada; 4 Women’s College Research Institute, Women’s College Hospital, Toronto, Canada; 5 Institute of Health Policy, Management and Evaluation, University of Toronto, Toronto, Canada; 6 Department of Medicine, University of Toronto, Toronto, Canada; 7 Leslie Dan Faculty of Pharmacy, University of Toronto, Toronto, Canada; 8 Center for Safety Research, SickKids Research Institute, Toronto, ON Canada

**Keywords:** Dosing, Error, Pediatrics, Critical care

## Abstract

**Background:**

There is no globally accepted definition for dosing error in adult or pediatric practice. The definition of pediatric dosing error varies greatly in the literature**.** The objective of this study was to develop a framework, informed by a set of principles, for a clinician-based definition of drug dosing errors in critically ill children, and to identify the range that practitioners agree is a dosing error for different drug classes and clinical scenarios.

**Methods:**

We conducted a nationwide three staged modified Delphi from May to December 2019. Expert clinicians included Canadian pediatric intensive care unit (PICU) physicians, pharmacists and nurses, with a least 5 years’ experience. Outcomes were underlying principles of drug dosing, and error thresholds, as defined by proportion above and below reference range, for common PICU medications and clinical scenarios.

**Results:**

Forty-four participants met eligibility, and response rates were 95, 86 and 84% for all three rounds respectively. Consensus was achieved for 13 of 15 principles, and 23 of 30 error thresholds. An over-dosed drug that is intercepted, an under-dose of a possibly life-saving medication, dosing 50% above or below target range and not adjusting for a drug interaction were agreed principles of dosing error. Altough there remained much uncertainty in defining dosing error, expert clinicians agreed that, for most medication categories and clinical scenarios, dosing over or below 10% of reference range was considered an error threshold.

**Conclusion:**

Dosing principles and threshold are complex in pediatric critical care, and expert clinicians were uncertain about whether many scenarios were considered in error. For most intermittent medications, dosing over 10% below or above reference range was considered a dosing error, although this was largely influenced by clinical context and drug properties. This consensus driven error threshold will help guide routine clinical dosing practice, standardized reporting and drug quality improvement in pediatric critical care.

## Background

Dosing errors are the amongst the most common types of medication error in pediatric practice, along with incomplete and incorrect prescription preparation [1–6]. .Pediatric drug dosing is particularly challenging due to a child’s growth, development and change in organ function over time. Dosing in children is therefore weight-based, involves the dilution of concentrated stock solutions, and the off-label use of medications approved for adults [7]. Despite standard weight-based dosing in pediatrics, doses used in clinical practice can vary between practitioners, clinical settings, and healthcare centers. Dosing is often based on patient characteristics and clinician comfort [8], due to a lack of established evidence based guidelines for appropriate pediatric dosing of off-label drugs in children. Critically ill children present a further challenge for dosing due to the pharmacokinetic changes associated with concurrent organ dysfunction and acuity of illness, as well as the pharmacodynamic properties associated with the concomitant exposure of other potentially harmful medications.

There is no consensus definition for dosing error in adult or pediatric practice [9]. Although a clinician-based definition for prescribing error has been proposed in pediatrics [8, 10], no such definition exists specifically exists for dosing error. Heterogenous definitions of pediatric dosing error in the literature include an “error in drug dosing, ordering, transcribing, administering or monitoring” [1, 4, 11, 12]; a dosing error “higher than reference dosing” [3]; “over 10% difference from reference dosing” [13]; “over 20% dose deviation from recommended dose” [7], +/− 25% of recommended dose (Ghaleb) or pharmacist consensus [14].

In clinical practice, critical care practitioners may prescribe acceptable doses outside a reference range, based on drug properties, organ dysfunction, and the patients’ clinical status. Dosing references and a more concrete dosing error definition are still needed however, to guide standardized error reporting, quality improvement for optimal prescribing practices, error comparisons within research and clinical decision support tools. We sought to better understand what constitutes a dosing error in the pediatric intensive care unit (PICU). The specific objectives of this study were 1) to develop a framework, informed by a set of principles, for a clinician-based definition of drug dosing errors in critically ill children, and 2) to identify the range, outside the standard reference range, that practitioners agree is a dosing error for different drug classes and clinical scenarios.

## Methods

### Design

We conducted a nationwide, three staged, modified Delphi method from May to December 2019, in Canada. We developed a detailed survey eliciting the opinions of PICU healthcare providers on 1) the underlying principles that guide dosing and influence the consideration of dosing error, and 2) dosing thresholds for dosing errors, in pediatric critical care. The study reporting followed the STROBE guidelines (STROBE checklist, Supplement Table 1) [15].

The Delphi methodology was used to achieve consensus amongst a group of PICU experts, with multiple iterative rounds and anonymous feedback after each round [16]. This methodology was favoured over other structured consensus methods as it did not require face-to-face group interaction, was questionnaire based, preserved anonymity of respondents, and has been frequently used and accepted for scientific consensus of this nature [17, 18]. Furthermore, the electronic distribution of the survey allowed for the timely completion of a nationwide survey with multiple rounds for participants. Given that this Delphi was conducted across Canada, we opted for a modified Delphi approach. A modified Delphi differs from the classical Delphi method in that the expert panel was not involved in the initial process of generating principles; this initial step was replaced by local face-to-face focus groups [19].

### Target population

We conducted deliberate purposive sampling of expert clinicians across Canada. Eligible clinicians were physicians, nurse managers and pharmacists who were currently working the majority of their time in a Canadian PICU, with at least 5 years PICU experience. Participants were contacted through the electronic mailing list of the Canadian Critical Care Trials Group (CCCTG), asking for their interest in participating. From this mailing list, we then further attempted to solicit at least one eligible physician, nurse and pharmacist from each center, by asking CCCTG members to refer participants who may be interested. Participation on the expert panel was voluntary. No incentives were offered.

An a priori sample size of 40 expert healthcare providers was established [8, 10, 17, 18]. This is slightly higher than previous pharmacological studies using Delphi consensus methodology. Assuming an 80% response rate and loss of adherence through three rounds, recruitment was maintained open until at least 50 providers agreed to participate.

### Outcomes

Two outcomes were assessed: dosing principles and error thresholds. Dosing principles were elicited by literature review and small local focus groups, and then measured by the expert panel with the strength of agreement and disagreement on a Likert scale. Error thresholds were defined as the proportion above and below reference range that expert clinicians agreed was a dosing error, given the specific drug class and clinical context.

### Intervention

#### Survey creation - item generation & reduction

The survey was generated using a guide for self-administered surveys of clinicians [20]. Survey principles were generated through literature review, and expert opinion in small local focus group sessions. Two small multidisciplinary focus groups were held with target clinicians (*n* = 9, including physicians, pharmacists and nurses) in order to brainstorm factors that may affect dosing threshold. Groups were continued until no new items were generated, and dosing threshold cut-offs were established. The rationale for the medication classes tested in the survey were based on the medications with the most frequent dosing errors in pediatrics; anti-infectives, analgesic and sedative agents, and electrolytes [1, 6, 7]. Item reduction was achieved by eliminating superfluous questions to minimize respondent burden, while retaining items that addressed a variety of medications classes, adjustment for organ dysfunction and pharmacokinetic properties. Further items were removed and/or altered after pre-testing the survey amongst clinicians. Error thresholds were established with focus group consensus and translated to a Likert scale (See Supplement Figure 1). Three of the nine clinicians from the focus groups went on to participate in the Delphi.

#### Survey format

The survey was divided into three sections; demographic description of respondents, dosing principles and error thresholds. Dosing principles included drug, patient and clinical factors and were scored on a 5-point Likert scale from “Yes, definitely an error” to “No, definitely not an error” (See Supplement Table 2). The dosing error thresholds were on a 4- or 5- point Likert scale. There were 4 thresholds categories for under-dosing (1–10%, 11–20%, 21–50%, 51–100%) and 5 for overdosing (1–10%, 11–20%, 21–50%, 51–100, > 100%). The option for “I don’t know” was always available to acknowledge uncertainty, as was the option “Depends” to identify new issues or principles that may suggest an error in one type of clinical scenario, but not another. An example of an error threshold round 2 question is included in Figure 1 in the Supplement.

#### Testing

Pre-testing of the survey was conducted with the help of clinicians in 2 different PICUs to aid in refining questionnaire content and the format of answers. Pilot testing, conducted in 2 centers, evaluated clinical sensibility including: face validity; content validity, whether questionnaire was measuring what it was intended to measure, clarity and comprehension.

#### Survey administration

Expert clinician participants were recruited via e-mail. Upon agreement to participate, an electronic survey or paper survey was sent, according to respondent preference and the desire to preserve anonymity. All surveys were anonymous and consent was implicit upon survey completion. Clinicians who responded but were ineligible, were not included in analysis or further rounds. Participants received 3 reminders to complete each round. In rounds 2 and 3 of the survey, questions where consensus was achieved were removed, and aggregated responses of participants were included for questions where consensus was not achieved.

### Data analysis

Data were summarized by numbers and proportions, and reported according to proposed methodological criteria for Delphi [21]. An a priori definition of consensus was established. Consensus was achieved when > = 70% of respondents agreed on an answer in Round 1, and > = 60% of respondents agreed to an answer in Round 2 and Round 3 [21]. Disagreement was defined as 35% or more of responses falling in both of the 2 extreme ranges of options on the Likert scale. All other combinations of panel answers were considered as ‘partial agreement’. Blank responses were entered as blank, and proportions were adjusted accordingly. Data from surveys were imported and analyzed with Excel version 16.2. Research Ethics Board Approval was obtained from the Hospital for Sick Children (# 1000062863) and the University of Toronto (#00037969).

## Results

**Fig. 1:**
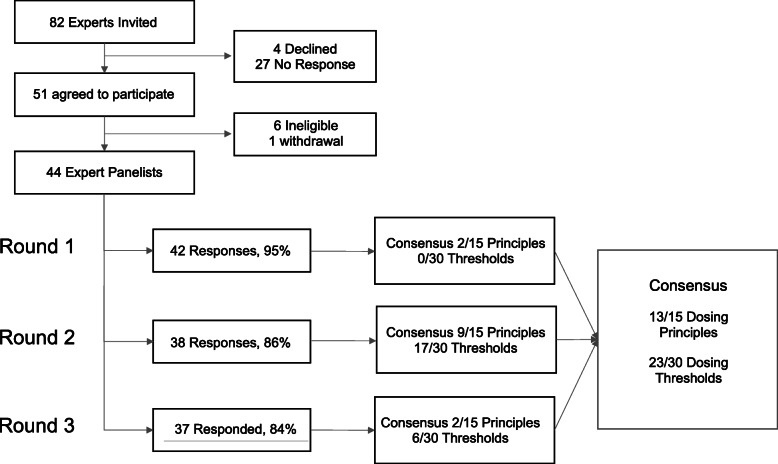
Flow chart of expert clinician participation in Delphi, and responses throughout rounds

We invited 82 clinicians to participate in the study, of which 51 (62%) agreed to participate. Of these, 44 (89%) providers met eligibility criteria, and were invited to complete further rounds. Figure 1 illustrates the flow chart of the modified Delphi process. Response rates for Rounds 1, 2 and 3 were 95, 86 and 84% respectively. Expert clinicians came from 14 pediatric centers in 6 Canadian provinces.

Of the 44 included expert clinicians, 42 completed round 1; 26 (62%) physicians, 13 (31%) pharmacists and 3 (7%) nurses. The providers prescribed medication occasionally (1–5 times/month) or daily in 11 and 26 cases, and administered medication never or occasionally (< 6 times per month) in 14 and 25 cases respectively. The demographic information on participants, for all three rounds, is included in Table 1.

**Table 1:** Demographics of included expert participants

	Round 1 *N* = 42, n (%)	Round 2 *N* = 38, n (%)	Round 3 *N* = 37, n (%)
**Healthcare Providers**			
Physician	26 (62)	23 (61)	24 (65)
Pharmacist	13 (31)	13 (34)	11 (30)
Nurse	3 (7)	2 (5)	2 (5)
**Length of Practice in PICU**			
5–10 years^a^	18 (43)	15 (39)	15 (41)
10–15 years	7 (17)	12 (32)	10 (27)
> 15 years	17 (40)	11 (29)	12 (32)
**Size of PICU**			
< 10 beds	3 (7)	3 (8)	3 (8)
10–15 beds	16 (38)	11 (19)	13 (35)
16–20 beds	7 (17)	8 (21)	5 (14)
21–25 beds	6 (14)	4 (10)	3 (8)
> 25 beds	10 (24)	12 (32)	13 (35)
**Province**			
British-Columbia	3 (7)	3 (8)	2 (5)
Alberta	3 (7)	2 (5)	2 (5)
Manitoba	4 (10)	2 (5)	3 (8)
Ontario	14 (33)	13 (34)	16 (43)
Quebec	17 (40)	17 (40)	13 (35)
Nova-Scotia	1 (2)	1 (3)	1 (3)

*PICU* Pediatric Intensive Care Unit

^a^1 participant with 5 years’ experience (including fellowship) was included

The most frequently reported resources used for pediatric dosing were: the local hospital formulary (*N* = 38), Lexi-comp on-line (N-34), memorized doses (*N* = 30), local hospital pharmacist (*N* = 29), and local ICU guidelines (*N* = 22). Less frequently used resources included the electronic Compendium of Pharmaceuticals and Specialties (e-CPS) (*N* = 4), UpToDate (*N* = 3), and PubMed (*N* = 1).

### Principles of dosing errors

Table 2 presents the 15 different patient-, drug- and dosing- principles evaluated, the agreement statement and the proportion of consensus agreement for each principle. Consensus was achieved for 13 of 15 principles: 2 principles were rated as “definite dosing error”, 3 were rated as “probable dosing error”, 7 as “may or may not be a dosing error”, 1 as “probably not a dosing error”, and 2 with partial agreement achieved. No principles were considered “definitely not dosing error”. An ‘over-dosed drug that is intercepted’, and an ‘administered under-dose of a possibly life-saving medication’, were agreed to be definite dosing errors. Experts agreed that ‘dosing 50% above or below target recommended range and not adjusting for a drug interaction’ was a probable dosing error. Experts also agreed on principles which may or may not constitute a dosing error; including ‘weight-based dosing without accounting for age, body surface area, renal insufficiency’, and ‘dosing above maximum recommended dosing’ (whether on Extracorporeal Membrane Oxygenation (ECMO), end of life, or neither). The only statement that experts felt was unlikely to be an error was ‘dosing a drug that cannot easily be administered in dosage forms available’. There were no statements that experts felt definitely did not constitute a dosing error. The principle addressing drug dosing after a previous adverse drug event (ADE), and the principle regarding off-label drug dosing, did not achieve consensus.

**Table 2:** Principles of dosing error, agreement statement, and proportion of consensus agreement^a^ for each statement

	Proportion of agreement (%) Round agreement achieved
**YES, DEFINITELY constitutes a dosing error**	
Dosing a drug above reference range, but the drug dose was intercepted (near-miss) and did not reach patient	**76** Round 1
Dosing a drug below the minimum recommended dose, when the drug is possibly life-saving	**73** Round 2
**YES, PROBABLY constitutes a dosing error**	
Dosing a drug in a dose that is predicted to give serum levels below desired therapeutic range (e.g. 50% below lower limit target)	**62** Round 2
Dosing a drug by weight, without accounting for a significant drug interaction	**68** Round 3
Dosing a drug in a dose that is likely to give serum levels above desired therapeutic range (e.g. 50% above upper limit target)	**60** Round 2
**MAY or MAY NOT be a dosing Error**	
Dosing a drug by weight, without adjusting for age	**76** Round 2
Dosing a drug by weight, without accounting for body surface area	**73** Round 1
Dosing a drug dose above the maximum dose recommended in the hospital formulary, guideline or reference sources	**66** Round 2
Dosing a drug above the maximum recommended dose, in a patient who is in end-of life care	**71** Round 2
Dosing a drug above the maximum recommended dose in a patient who is on ECMO^b^	**82** Round 2
Dosing a drug above the maximum recommended dose, when the drug is possibly life-saving (e.g. antibiotics in sepsis)	**60** Round 2
Dosing a drug by weight, without adjusting for renal insufficiency (Creatinine 50% higher than baseline)	**69** Round 3
**NO, UNLIKELY to constitute a dosing error**	
Dosing a drug dose that cannot easily be administered using dosage forms available	**66** Round 2
**NO, DEFINITELY NOT a dosing error**	
None	
Dosing a drug at a dose where the patient has had a previous severe adverse event	Partial agreement^c^
Dosing a drug for which the use is off-label in children (i.e. prescribed for a condition that it is not officially approved for)	Partial agreement^d^

N for Round 1 is 42, N for Round 37, N for Round 3 is 36

^a^Consensus agreement established a priori was > = 70% for Round 1, and > =60% for Rounds 2 and 3.

^b^ECMO: Extra-Corporeal Membrane Oxygenation.

^c^*N* = 21 (58%) responded “Yes, definitely dosing error”, *N* = 1 “Yes, probable dosing error” and *N* = 14 (39%) “May or May not be error”. N total = 36 participants.

^d^*N* = 20 (55%) “No Definitely not a dosing error”, *N* = 12 (33%) “No, probably not a dosing error”, *N* = 3 (8%) “May or may not be a dosing error”, *N* = 1 (3%) “I don’t know”. N total = 36 participants.

### Error thresholds

We evaluated 15 above and below dosing error thresholds (*N* = 30 error thresholds). Consensus was achieved for 23 thresholds; 12 under-dose error thresholds and 11 over-dose error thresholds. Progression of the consensus over 3 rounds is presented in Fig. 1. Where consensus was achieved, expert providers agreed, for most medication categories and clinical scenarios that dosing over or below 10% of reference range was considered an error (category 11–20% was an error). Results of the error thresholds are presented in Table 3.

**Table 3:** Proportion below and above reference range dosing that expert participants would consider is a dosing error, and proportion of consensus agreement

Drug Class and Patient characteristics	Threshold for dosing error below reference range (% consensus)	Threshold for dosing error Above reference range (% consensus)
**NEPHROTOXIC ANTIBIOTIC** (e.g. Gentamicin, Vancomycin)		
In a patient with normal renal function^a^	10% (81)	10% (79)
In a patient with acute renal failure^b^	10% (76)	10% (74)
**NON-TOXIC ANTIBIOTIC** (e.g. Ampicillin)^a^		
In stable patient, no organ dysfunction	10% (68)	Partial agreement^c^
**HEPATOTOXIC MEDICATION** (e.g. Acetaminophen)		
In patient with normal hepatic function	10% (74)	10% (76)
In a patient with acute hepatic failure	10% (76)	10% (74)
**OPIOID** (e.g Morphine)		
In an opioid naïve patient with normal organ function	10% (66)	10% (71)
In a patient with an opioid tolerance	10% (63)	Partial agreement^d^
In a patient with acute renal failure^b^	10% (74)	10% (79)
In a patient with a previous opioid related adverse event^h^	Partial agreement^e^	Partial agreement^e^
**BENZODIAZEPINE** (e.g. Lorazepam)		
In an opioid naïve patient with normal organ function	10% (61)	10% (79)
In a patient with acute renal failure^b^	10% (66)	10% (84)
In a patient with a previous opioid related adverse event^h^	Partial agreement^f^	10% (60)
**ANTICOAGULANT** (e.g. Heparin)		
In a stable patient, no organ dysfunction	10% (71)	10% (73)
In a patient with a previous heparin-related hemorrhage	Partial agreement^g^	1% (71)
**ELECTROLYTE BOLUS** (e.g. Potassium chloride)		
In stable patient, no organ dysfunction	10% (66)	Partial agreement^i^

^a^Assume moderate infection, normal hepatic function and normal BMI. No drug levels yet.

^b^Assume moderate infection, normal hepatic function and normal BMI. No drug levels yet. Renal dosing adjustment with Creatinine 2x normal

^c^*N* = 20 suggested > 20% for upper threshold, and *N* = 12 suggested > 10%, *N* = 4 blank. N total = 36.

^d^*N* = 19 suggested > 10% for upper threshold, and *N* = 7 suggested > 10%, *N* = 5 > 50%. N total = 36.

^e^*N* = 6 would not give drug at all. For overdoing, *N* = 22 (58%) answered 1–10%, *N* = 7 (18%) answered > 10%, *N* = 1 answered> 20%, *N* = 1 blank.

For under-dosing, *N* = 18 (47%) answered 21–50%, *N* = 6 (16%) answered 11–20%.

^f^*N* = 6 would not give drug at all, *N* = 14 suggested > 10% for upper threshold, and *N* = 9 suggested > 10%.

^g^*N* = 3 (8%) answered 1–10%, *N* = 21 answered > 10% (58%), *N* = 7 answered > 20% (19%), *N* = 2 answered > 50%, *N* = 4 blank or I don’t know. N total = 37.

^h^Adverse drug event occurred with the drug dose in reference range, and resulted in a 2-day prolongation of hospital stay.

^i^*N* = 12 answered > 10% (32%), *N* = 21 answered > 20% (57%), *N* = 1 answered > 51%, *N* = 3 blank. N total = 37.

Consensus not achieved for 2/15 principles.

For nephrotoxic antibiotics (such as gentamicin and vancomycin), 10% above and below reference range, when renal function was adjusted for, was considered an error. For hepatotoxic medications (such as acetaminophen and NSAIDs), 10% above and below reference range, when hepatic function was adjusted for, was also considered an error. Error thresholds for opioid and benzodiazepines over-dose error were 10% above reference range regardless of presence of renal failure, as long as dose was renally adjusted. When the scenario included opioid tolerance there was consensus on the lower threshold but only partial agreement on the upper threshold for dosing (dosing > 10% remained the most common choice *n* = 19 (50%)).

For non-toxic antibiotics (such as Ampicillin), partial agreement of over 50% expert providers considered > 20% above reference range as an error and 10% below reference to be an error (consensus).

In all three scenarios where a previous adverse drug event had occurred with the same drug, experts never achieved consensus on the upper threshold for error, and were more conservative in their dosing. In a number of cases (*N* = 6), clinicians said they would not give the drug to a patient with a previous serious adverse drug event. Many others answered ‘Depends’ and suggested that the circumstances of the ADE where important to consider: nature and severity of the ADE, whether it was allergic or not, the concomitant medications, and the balance of desirable versus undesirable side effects. This finding is consistent with the answers on the dosing principles as consensus was also not achieved when evaluating a dose where a previous ADE has occurred. In medications with a high potential toxicity (e.g. potassium chloride), clinicians were also unwilling to exceed reference dosing (partial agreement, *N* = 21/37 (56%)).

Sixty-six percent of clinicians said over-dosing was not equivalent to under-dosing; and this was justified in comments mostly relating to the effects of over-dose vs under-dose.

## Discussion

This study used a modified Delphi methodology to establish a framework of principles and dosing error thresholds for drugs commonly given in the PICU under common clinical scenarios. A group of 44 PICU clinicians were recruited from across Canada with participation in all three rounds exceeding 80%. Clinicians agreed that under-dosing potentially life-saving drugs in the PICU is a definite error, as were near-miss over-doses. Under-dosing and over-dosing expected to give low or high serum concentration was also a probable error. There were many scenarios however, where clinicians agreed that establishing error was more difficult and complex. These scenarios include weight-based dosing without accounting for age, body surface area, or renal dysfunction, and dosing above the maximum limit in ECMO or end-of-life.

Expert PICU clinicians agreed that, for most drugs in most clinical scenarios, dosing more than 10% above and below reference range was an error threshold. This was internally consistent with the principle of over and under-dosing described above, as well as commonly accepted practice amongst many pharmacists. There were however, scenarios where defining error was difficult despite consensus; including adjusting dosing for age, body mass index, renal impairment, and exceeding maximum dosing on ECMO, in end of life care or if the drug is ‘life saving’ (Table 2). The consensus achieved in the ambiguity of defining these scenarios as errors, reflects the complexity that prescriber experience, intent and patient clinical context may render excess dosing as acceptable. There were other scenarios where consensus could not be reached; including all clinical scenarios where a previous ADE had occurred. Although some providers adjusted their error thresholds to more narrow dosing, others simply would not administer the drug after a harmful ADE.

Responses in dosing error thresholds were mostly consistent with the consensus on the dosing principles. There were however, scenarios where clinicians were not completely consistent in their choices: they agreed that ‘dosing 50% above or below target range and not adjusting for a drug interaction’ was a probable dosing error, despite the fact that most chose a 10% overdose threshold. The choice of ‘probable’, ‘definite’ and ‘may or may not be’ a dosing error, suggests that clinicians were able to potentially conceive of instances where selective overdosing might not be an error, and reflects the complexity of defining error.

Despite clinicians selecting the same thresholds for many medication groups and clinical scenarios, they were able to modify and adapt their responses to different clinical scenarios. This adaptability is evidenced by the lower threshold selected for the anticoagulant dosing with previous adverse event (1% threshold versus 10% when ADE had occurred).

The results of this study reflect the complexity of dosing in clinical practice and the difficulty in establishing a definition. Participants were uncertain as to whether many clinical scenarios constituted dosing errors (Table 2). That being said, there is a need for a more pragmatic definition of dosing error. Drug dosing error evaluation (causes, etiology, related adverse events) first requires defining whether an error has occurred to establish preventability; adverse events may occur with an appropriately dosed drug, or with an inappropriately dosed drug (under- or over-dosed). Although Ghaleb et al. evaluated principles of prescription error in pediatrics [8], there definition contains subjective terms related to the significance and harm related to an error. Within regards to dosing, consensus was achieved amongst their participants that dosing a drug above or below 25% of the reference range should be an error [8]. A recent pediatric study by Howlett et al. used a Delphi method to identify medication errors related the health information systems, but these did not address dosing ranges [18]. The current heterogeneity in defining dosing error in the literature makes comparison of results across studies of medication error very difficult, and fails to adequately define optimal prescribing practices. This study will help define dosing error thresholds for the prescription of intermittent drugs in routine clinical practice and future research studies, as defined by expert specialists -filling a gap in the pediatric dosing literature. These consensus-based results may facilitate measurement and research comparisons, along with providing guidance for quality improvement initiatives and optimal prescribing, standardized error reporting, and building decision support tools with health system informatics.

This study has a few limitations. First, participants in this study may not adequately reflect other PICU providers, both within and outside Canada - therefore generalizability may be limited. That being said, limited pediatric dosing resources and definitions, as well as our use of proportions rather than absolute values, will likely make these dosing principles and thresholds clinically valid internationally. Second, we could not exclude neutral response bias of clinicians selecting the same response throughout. Questionnaire burden was a significant concern, given the multiplicity of rounds and survey length. Although we reached over 80% response rate for each round, study participation took 10–15 min over the 3 different rounds and required active participation to send back the surveys. We therefore deliberately limited the medication groups and clinical scenarios that were included to limit the respondent burden. Third, the study did not assess continuous infusions of sedatives, analgesics or inotropes commonly used in the PICU, nor did it address rate of administration of intermittent drugs. Fourth, the low nursing participation in the study (*n* = 4) unfortunately limited the weight of nursing respondents in this study. Although most nurses do not prescribe medications in the PICU, they are largely responsible for drug administration. Understanding the subtleties of drug dilution, preparation and administration, their opinions on dose errors would have provided valuable input. Last, although dosing error is made up of prescribing error, dispensing error and administration error; this manuscript does not specifically distinguish between these errors, nor does it address the intentionally of the prescriber. We focused most specifically on administered drug to the patient, i.e. the dose the patient receives, whether the deviation occurred during prescription, preparation or administration was not addressed but may have changed response selection. Pharmacy convention however, suggests that a less than 10% dose deviation during preparation may be acceptable. Prescriber intent further complexifies defining error. If an experienced provider deliberately doses outside refence range and this is not considered an error, then that extended dosing should be reflected in the acceptable principles and thresholds tested in this study (i.e. clinicians can conceive of these acceptable deviations in dosing).

Overall, the consensus driven clinical dosing principles and error thresholds provides useful clinical data to begin to define dosing error in pediatric critical care practice. Further studies are needed to test and refine this 10% dosing threshold in critical care; including studies of association between 10% excess dosing and adverse drug events (harm), and prospective studies using adjudication to asses acceptable dosing for different drug classes in various clinical contexts.

## Conclusion

This study provides the first generalizable expert consensus on dosing principles and dosing thresholds in pediatrics. Expert clinical providers in the PICU agreed on the uncertainty in what underlying principles constitute a dosing error, reflecting the complexity in defining it. For most medications and clinical scenarios, dosing over 10% below or above reference range was considered a dosing error. However, clinical context and drug properties largely influences this threshold. Thresholds may be larger for non-toxic medications, and narrower for very toxic medications, medications with larger side effects or if a previous adverse drug event occurred. These consensus driven findings will help guide prescribing practice, standardized reporting, measurement comparisons in research and drug quality improvement in pediatric critical care.

## Supplementary information

**Additional file 1: Table S1.** STROBE Checklist. **Table S2.** Principles of dosing error included in Delphi questionnaire. **Figure S1.** Example of dosing error threshold question for Round 2.

## Data Availability

The datasets generated and/or analysed during the current study are not publicly available, but available from the corresponding author [NR] on request.

## Abbreviations

ADE: Adverse Drug Events
CCCTG: Canadian Critical Care Trials Group
ECMO: Extracorporeal Membrane Oxygenation
NSAIDs: Non-Steroidal Anti-Inflammatory Drugs
PICU: Pediatric Intensive Care Unit
