# Supplementary material for: A modified Delphi to define drug dosing errors in pediatric critical care
Source: BMC Pediatr. 2020 Oct 21;20:488. doi: 10.1186/s12887-020-02384-3 (PMC7576795; doi:10.1186/s12887-020-02384-3)
Supplement: Supplementary file 1 — Additional file 1: Table S1. STROBE Checklist. Table S2. Principles of dosing error included in Delphi questionnaire. Figure S1. Example of dosing error threshold question for Round 2. [file 12887_2020_2384_MOESM1_ESM.zip › FIGURES_SUPPLEMENTR1.docx]

Figure 2.

| **Drug X is a NEPHROTOXIC ANTIBIOTIC** |
| --- |
| In a patient with normal renal function, what proportion of Drug X dose, would you consider is a dosing error? |

N=38, Consensus on Round 2

| **Drug X is a NEPHROTOXIC ANTIBIOTIC** |
| --- |
| If your patient has acute renal failure, and you have dose adjusted Drug X to a new renally-adjusted reference range, what proportion of Drug X would you consider is a dosing error? |

N=38 for Below reference range, consensus on Round 2, N-37 for Above reference range, Consensus on Round 3

| **DRUG X is a NON-TOXIC ANTIBIOTIC**  What **proportion of Drug X dose,** above or below reference, would you consider is a dosing error? |
| --- |

N=38 below reference range consensus in Round 2, N-37 for above reference range, no consensus

| **DRUG X is a HEPATOTOXIC MEDICATION** |
| --- |
| 4. What **proportion of Drug X dose,** above or below reference, would you consider is a dosing error? |

N=38, Consensus achieved Round 2

| **DRUG X is an OPIOID** |
| --- |
| 6. What **proportion of Drug X dose,** above or below reference, would you consider is a dosing error? |

N=38, Consensus in Round 2

| **DRUG X is an OPIOID** |
| --- |
| 7. If the patient had an opioid tolerance in the PICU, what proportion of an intermittent single dose of Drug X would you consider is a dosing error? |

N=37, Consensus for Below Reference achieved in Round 2

| **DRUG X is an OPIOID** |
| --- |
| 8. If your patient has acute renal failure, what proportion of Drug X would you consider is a dosing error? |

N=38 for Below reference range (Consensus Round 2), and N=37 for Above reference range (Consensus Round 3).

| **DRUG X is an OPIOID** |
| --- |
| 9. If the patient has had a previous adverse event related to an opioid within reference range, what proportion of Drug X would you consider is a dosing error? |

N=30 for below and N=31 for above. N=6 selected “I would not give this drug”.

| **DRUG X is a BENZODIAZEPINE** |
| --- |
| 10. What proportion of Drug X would you consider is a dosing error? |

N=38 Consensus in Round 2.

| **DRUG X is a BENZODIAZEPINE** |
| --- |
| 11. If your patient has acute renal failure, what proportion of Drug X would you consider is a dosing error? |

N=37, Consensus in Round 2. N=1 said I don’t know.

| **DRUG X is a BENZODIAZEPINE** |
| --- |
| 12. If the patient has had a previous adverse event related to an opiood within reference range, what proportion of Drug X would you consider is a dosing error? |

N=37 respondents. N=6 said “I would not give this drug”

| **DRUG X is a ANTICOAGULANT** |
| --- |
| 13. What proportion of Drug X would you consider is a dosing error? |

N=38 for Below Reference Range. Consensus in Round 2. , N=38 for above reference range, Consensus in Round 3.

| **DRUG X is a ANTICOAGULANT** |
| --- |
| 14. A decision is made to anticoagulate a patient with heparin, but h/she has had a previous heparin related hemorrhage 1 month ago. What proportion of an initial dose of Heparin would you consider is a dosing error? |

N=38 for Below reference range, N=36 for Above reference range (N=1 say “I don’t know”)

| **DRUG X is an ELECTROLYTE BOLUS** |
| --- |
| 15. What proportion of Drug X would you consider is a dosing error? |

N=38 for Below reference range, N=36 for Above reference range
