# Supplementary material for: A modified Delphi to define drug dosing errors in pediatric critical care
Source: BMC Pediatr. 2020 Oct 21;20:488. doi: 10.1186/s12887-020-02384-3 (PMC7576795; doi:10.1186/s12887-020-02384-3)
Supplement: Supplementary file 1 — Additional file 1: Table S1. STROBE Checklist. Table S2. Principles of dosing error included in Delphi questionnaire. Figure S1. Example of dosing error threshold question for Round 2. [file 12887_2020_2384_MOESM1_ESM.zip › SUPPLEMENTR1.docx]

SUPPLEMENT

A Modified Delphi to Define Drug Dosing Errors in Pediatric Critical Care

Table 1. STROBE Checklist Page 2-3

Table 2. Principles of dosing error included in Delphi questionnaire Page 4

Figure 1. Example of dosing error threshold question for Round 2 Page 5

Figures 2. A-G. Final Consensus Figures for Dosing Thresholds Page 6-12

1. Nephrotoxic Antibiotics
2. Non-Toxic Antibiotics
3. Hepatotoxic medication
4. Opioids
5. Benzodiazepines
6. Anticoagulants
7. Electrolyte Bolus

Table 1.STROBE Statement—Checklist of items that should be included in reports of ***cohort studies***

|  | Item No | Recommendation | Page No |
| --- | --- | --- | --- |
| **Title and abstract** | 1 | (*a*) Indicate the study’s design with a commonly used term in the title or the abstract | 1-2 |
|  |  | (*b*) Provide in the abstract an informative and balanced summary of what was done and what was found |  |
| Introduction | | | |
| Background/rationale | 2 | Explain the scientific background and rationale for the investigation being reported | 4 |
| Objectives | 3 | State specific objectives, including any prespecified hypotheses | 4 |
| Methods | | | |
| Study design | 4 | Present key elements of study design early in the paper | 5 |
| Setting | 5 | Describe the setting, locations, and relevant dates, including periods of recruitment, exposure, follow-up, and data collection | 5-6 |
| Participants | 6 | (*a*) Give the eligibility criteria, and the sources and methods of selection of participants. Describe methods of follow-up | 5 |
|  |  | (*b*) For matched studies, give matching criteria and number of exposed and unexposed |  |
| Variables | 7 | Clearly define all outcomes, exposures, predictors, potential confounders, and effect modifiers. Give diagnostic criteria, if applicable | 6 |
| Data sources/ measurement | 8* | For each variable of interest, give sources of data and details of methods of assessment (measurement). Describe comparability of assessment methods if there is more than one group | 5 |
| Bias | 9 | Describe any efforts to address potential sources of bias | 7 |
| Study size | 10 | Explain how the study size was arrived at | 6 |
| Quantitative variables | 11 | Explain how quantitative variables were handled in the analyses. If applicable, describe which groupings were chosen and why | 7 |
| Statistical methods | 12 | (*a*) Describe all statistical methods, including those used to control for confounding | 7-8 |
|  |  | (*b*) Describe any methods used to examine subgroups and interactions |  |
|  |  | (*c*) Explain how missing data were addressed |  |
|  |  | (*d*) If applicable, explain how loss to follow-up was addressed |  |
|  |  | (*e*) Describe any sensitivity analyses |  |
| Results | | |  |
| Participants | 13* | (a) Report numbers of individuals at each stage of study—eg numbers potentially eligible, examined for eligibility, confirmed eligible, included in the study, completing follow-up, and analysed | Figure1 |
|  |  | (b) Give reasons for non-participation at each stage |  |
|  |  | (c) Consider use of a flow diagram |  |
| Descriptive data | 14* | (a) Give characteristics of study participants (eg demographic, clinical, social) and information on exposures and potential confounders | Table 1 |
|  |  | (b) Indicate number of participants with missing data for each variable of interest |  |
|  |  | (c) Summarise follow-up time (eg, average and total amount) |  |
| Outcome data | 15* | Report numbers of outcome events or summary measures over time | 9-11 |

| Main results | 16 | (*a*) Give unadjusted estimates and, if applicable, confounder-adjusted estimates and their precision (eg, 95% confidence interval). Make clear which confounders were adjusted for and why they were included | N/A |
| --- | --- | --- | --- |
|  |  | (*b*) Report category boundaries when continuous variables were categorized |  |
|  |  | (*c*) If relevant, consider translating estimates of relative risk into absolute risk for a meaningful time period |  |
| Other analyses | 17 | Report other analyses done—eg analyses of subgroups and interactions, and sensitivity analyses | N/A |
| Discussion | | | |
| Key results | 18 | Summarise key results with reference to study objectives | 11--12 |
| Limitations | 19 | Discuss limitations of the study, taking into account sources of potential bias or imprecision. Discuss both direction and magnitude of any potential bias | 12 |
| Interpretation | 20 | Give a cautious overall interpretation of results considering objectives, limitations, multiplicity of analyses, results from similar studies, and other relevant evidence | 12 |
| Generalisability | 21 | Discuss the generalisability (external validity) of the study results | 12-13 |
| Other information | | | |
| Funding | 22 | Give the source of funding and the role of the funders for the present study and, if applicable, for the original study on which the present article is based | 3 |

**Table 2. Principles of Dosing Error included in Delphi questionnaire**

**Section II: PRINCIPLES OF DOSING ERROR IN PEDIATRIC CRITICAL CARE**

Please indicate whether the situation below constitutes a dosing error, in your opinion. Place **one** check mark per line.

|  | **Yes definitely** a dosing error | **Yes** **probably**  a dosing error | **May or may not** be a dosing error | **No**  **unlikely**  a dosing error | **No definitely** not a dosing error | **I do not know** |
| --- | --- | --- | --- | --- | --- | --- |
| **Patient Level Factors** |  |  |  |  |  |  |
| Dosing a drug by weight, without adjusting for renal insufficiency (Creatinine 50% higher than baseline) |  |  |  |  |  |  |
| Dosing a drug by weight, without accounting for body surface area |  |  |  |  |  |  |
| Dosing a drug by weight, without adjusting for age |  |  |  |  |  |  |
| Dosing a drug by weight, without accounting for a significant drug interaction |  |  |  |  |  |  |
| Dosing a drug at a dose where the patient has had a previous severe adverse event |  |  |  |  |  |  |
| **Drug Level Factors** |  |  |  |  |  |  |
| Dosing a drug for which the use is off-label in children (i.e. prescribed for a condition that it is not officially approved for) |  |  |  |  |  |  |
| Dosing a drug dose that cannot easily be administered using dosage forms available (eg: 2 mg Omeprazole, tablet is 7.5mg) |  |  |  |  |  |  |
| Dosing a drug in a dose that is likely to give serum levels above desired therapeutic range (eg. 50% above upper limit target) |  |  |  |  |  |  |
| Dosing a drug in a dose that is predicted to give serum levels below desired therapeutic range (50% below lower limit target) |  |  |  |  |  |  |
| Dosing a drug dose above the maximum dose recommended in the hospital formulary, guideline or reference sources |  |  |  |  |  |  |
| **Clinical Factors** |  |  |  |  |  |  |
| Dosing a drug **above** reference range (in toxic range), but the drug dose was intercepted (near-miss event) and did not reach patient |  |  |  |  |  |  |
| Dosing a drug **above** the maximum recommended dose, in a patient who is in end-of life care |  |  |  |  |  |  |
| Dosing a drug **below** the minimum recommended dose, when the drug is possibly life-saving (eg: antibiotics in sepsis) |  |  |  |  |  |  |
| Dosing a drug **above** the maximum recommended dose, when the drug is possibly life-saving (eg: antibiotics in sepsis) |  |  |  |  |  |  |
| Dosing a drug **above** the maximum recommended dose in a patient who is in on ECMO* |  |  |  |  |  |  |

Figure 1. Example of Dosing Threshold question in Round 2

Consider **Drug X is a nephrotic antibiotic** (e.g. Gentamycin or Vancomycin)

In a stable PICU patient with normal renal function, what **proportion of Drug X dose,** above or below reference range, would you consider is a dosing error?

Assume moderate infection, normal hepatic function and normal BMI. No drug levels yet.

To respond please place 2 check marks, one for under-dosing and one for overdosing.

Responses from the previous round of questions are provided in grey [N (%), N total=42].

| 0 | 9 (21) | 21 (50) | 7 (17) |  | 7 (17) | 22 (52) | 8 (19) | 0 | 1 (2) |
| --- | --- | --- | --- | --- | --- | --- | --- | --- | --- |
| 51-100% | 21-50%  (up to 2-fold decrease) | 11-20% | 1-10% | **Reference range** | 1-10% | 11-20% | 21-50% | 51-100% | >100%  (over 2-fold increase) |
| 🡨 Below reference range | | | |  | Above reference range 🡪 | | | | |

□ I don’t know [1 (2)]

□ Depends on the patient [10 (24)]

If you answered ‘depends on the patient’, please clarify

Figure 2. Responses for dosing thresholds in the different drug categories and clinical scenarios.
